# Supplementary material for: Fasting shapes chromatin architecture through an mTOR/RNA Pol I axis
Source: Nat Cell Biol. 2024 Sep 19;26(11):1903–17. doi: 10.1038/s41556-024-01512-w (PMC11567895; doi:10.1038/s41556-024-01512-w)
Supplement: Supplementary file 1 — Reporting Summary [file 41556_2024_1512_MOESM1_ESM.pdf]

nature portfolio

Corresponding author(s): Daphne S. Cabianca

Last updated by author(s): Aug 5, 2024

Reporting Summary

Nature Portfolio wishes to improve the reproducibility of the work that we publish. This form provides structure for consistency and transparency in reporting. For further information on Nature Portfolio policies, see our Editorial Policies and the Editorial Policy Checklist.

Statistics

For all statistical analyses, confirm that the following items are present in the figure legend, table legend, main text, or Methods section.

n/a	Confirmed	
☐	☒	The exact sample size (*n*) for each experimental group/condition, given as a discrete number and unit of measurement
☐	☒	A statement on whether measurements were taken from distinct samples or whether the same sample was measured repeatedly
☐	☒	The statistical test(s) used AND whether they are one- or two-sided<br>*Only common tests should be described solely by name; describe more complex techniques in the Methods section.*
☒	☐	A description of all covariates tested
☐	☒	A description of any assumptions or corrections, such as tests of normality and adjustment for multiple comparisons
☐	☒	A full description of the statistical parameters including central tendency (e.g. means) or other basic estimates (e.g. regression coefficient) AND variation (e.g. standard deviation) or associated estimates of uncertainty (e.g. confidence intervals)
☐	☒	For null hypothesis testing, the test statistic (e.g. *F*, *t*, *r*) with confidence intervals, effect sizes, degrees of freedom and *P* value noted<br>*Give P values as exact values whenever suitable.*
☒	☐	For Bayesian analysis, information on the choice of priors and Markov chain Monte Carlo settings
☒	☐	For hierarchical and complex designs, identification of the appropriate level for tests and full reporting of outcomes
☐	☒	Estimates of effect sizes (e.g. Cohen's *d*, Pearson's *r*), indicating how they were calculated

Our web collection on statistics for biologists contains articles on many of the points above.

Software and code

Policy information about availability of computer code

Data collection	Images were collected with a Confocal Spinning Disk Microscopefrom Visitron Systems GmbH, equipped as follows: Nikon Eclipse Ti2 microscope + Plan Apo λ 100X/1.45 oil objective, Plan apo λ 60X/1.40 oil objective, Yokogawa CSU-W1 confocal scanner unit, VS-Homogenizer, EMCCD camera [Andor - iXon Series], and VisiView software (version 5.0.0.17) for acquisition.<br>Images were also captured with a Leica M165 FC fluorescent stereo microscope connected to a Leica K5 camera, using the LAS X software.<br>RNA-seq data were collected using a NextSeq2000 (Illumina) sequencer.<br>qPCR data were collected on a LightCycler 96 (Roche).
Data analysis	Fiji/ImageJ 1.53c<br>ImageJ plugin PointPicker 578 (http://bigwww.epfl.ch/ thevenaz/pointpicker/)<br>Cell-ACDC (Padovani et al. BMC Biol. 2022)<br>Custom algorithms, code are available on GitHub at the following links:<br>https://github.com/SchmollerLab/SeelMito<br>https://github.com/ElpadoCan/ChromRings<br><br>RNAseq data were analyzed using the R packages QuasR v1.42.1, The EdgeR package v4.0.14, gprofiler2 package v0.2.2, bowtie2 v2.4.5 and Trimmomatic v0.39 and BSgenome. Celegans.UCSC.ce10<br>Statistics analysis (other than RNAseq data) were calculated in R (4.3.1) or Excel (Version 2308)<br>Plots were made in Python (3.10), Excel (Version 2308), R (4.3.1), GraphPad Prism (8.0.1) or gprofiler2 package v0.2.2.

For manuscripts utilizing custom algorithms or software that are central to the research but not yet described in published literature, software must be made available to editors and reviewers. We strongly encourage code deposition in a community repository (e.g. GitHub). See the Nature Portfolio guidelines for submitting code & software for further information.

Data

Policy information about availability of data

All manuscripts must include a data availability statement. This statement should provide the following information, where applicable:

- Accession codes, unique identifiers, or web links for publicly available datasets
- A description of any restrictions on data availability
- For clinical datasets or third party data, please ensure that the statement adheres to our policy

The RNA-seq datasets comparing fed and fasted worms have been deposited at GEO and can be accessed under GSE268926. The RNA-seq datasets measuring the effect of RNA-Pol I depletion have been deposited at GEO and can be accessed under GSE268974. The RNA-seq datasets analyzing the transcriptome following RPB-2 degradation have been deposited at bioproject and can be accessed under PRJNA1140129. Publicly available dataset used in this study: BSgenome. Celegans.UCSC.ce10 “https://bioconductor.org/packages/BSgenome.Celegans.UCSC.ce10/”
Source data have been provided in Source Data. All other data supporting the findings of this study are available from the corresponding author on reasonable request.

Research involving human participants, their data, or biological material

Policy information about studies with human participants or human data. See also policy information about sex, gender (identity/presentation), and sexual orientation and race, ethnicity and racism.

Reporting on sex and gender	N/A
Reporting on race, ethnicity, or other socially relevant groupings	N/A
Population characteristics	N/A
Recruitment	N/A
Ethics oversight	N/A

Note that full information on the approval of the study protocol must also be provided in the manuscript.

Field-specific reporting

Please select the one below that is the best fit for your research. If you are not sure, read the appropriate sections before making your selection.

☒ Life sciences ☐ Behavioural & social sciences ☐ Ecological, evolutionary & environmental sciences

For a reference copy of the document with all sections, see nature.com/documents/nr-reporting-summary-flat.pdf

Life sciences study design

All studies must disclose on these points even when the disclosure is negative.

Sample size	No statistical methods were used to predetermine sample size. Every experimental repetition included parallel imaging of multiple worms, resulting in samples sizes of mostly in the range of tens to hundreds foci, nuclei or nucleolus scored, as commonly done in the field (all n and N values are in Supplementary Table 2). Therefore, sample sizes were chosen based on previous literature & what is common practice in the field :<br>- Meister P, Towbin BD, Pike BL, Ponti A, Gasser SM. The spatial dynamics of tissue-specific promoters during C. elegans development. Genes Dev. 2010 Apr 15;24(8):766-82. doi: 10.1101/gad.559610. PMID: 20395364; PMCID: PMC2854392<br>- Cabianca DS, Muñoz-Jiménez C, Kalck V, Gaidatzis D, Padeken J, Seeber A, Askjaer P, Gasser SM. Active chromatin marks drive spatial sequestration of heterochromatin in C. elegans nuclei. Nature. 2019 May;569(7758):734-739. doi: 10.1038/s41586-019-1243-y. Epub 2019 May 22. PMID: 31118512
Data exclusions	No data was excluded
Replication	Every experiment was reliably reproduced with 2 to 5 biological replicates, as specified for each experiment in the figure legends or methods
Randomization	No method of randomization was used as this is not relevant to the field of study. All samples were allocated to a group according to their genotype or treatment. However, individual worms were selected randomly for each experiment.
Blinding	Investigators were not blinded to group allocation of samples (genotype/treatment) during most data collection and analysis. However, key

Blinding	findings (chromatin reorganization in fasting, RNA Pol I-AID) were reproduced by at least an additional independent investigator who was blinded to group identity. For qPCR, the investigator was blinded to group identity. For RNA-seq, correlation and clustering was performed for all samples and replicas at the same time, regardless of group allocation. After that, the investigator grouped the samples according to genotype/treatment to be able to average the results across 4 independent biological replicas.

Reporting for specific materials, systems and methods

We require information from authors about some types of materials, experimental systems and methods used in many studies. Here, indicate whether each material, system or method listed is relevant to your study. If you are not sure if a list item applies to your research, read the appropriate section before selecting a response.

Materials & experimental systems

n/a	Involved in the study	
☒	☐	Antibodies
☒	☐	Eukaryotic cell lines
☒	☐	Palaeontology and archaeology
☐	☒	Animals and other organisms
☒	☐	Clinical data
☒	☐	Dual use research of concern
☒	☐	Plants

Methods

n/a	Involved in the study	
☒	☐	ChIP-seq
☒	☐	Flow cytometry
☒	☐	MRI-based neuroimaging

Animals and other research organisms

Policy information about studies involving animals; ARRIVE guidelines recommended for reporting animal research, and Sex and Gender in Research

Laboratory animals	Caenorhabditis elegans (variant Bristol) hermaphrodites were used in this study. The developmental stages used are indicated in the manuscript (mostly L1s and 1 day old adults).<br>The strains (involved in the study are listed in Supplementary Table 1, together with their exact genotypes.
Wild animals	No wild animals were used in this study
Reporting on sex	Hermaphrodite were used for all experiments.<br>Males were used only to generate new strains via crossing.
Field-collected samples	This study did not involve samples collected from the field
Ethics oversight	As a non-vertebrate, C. elegans does not fall under the Directive 2010/63/EU of the European Parliament and of the Council of 22 September 2010 on the protection of animals used for scientific purposes:<br>http://eur-lex.europa.eu/LexUriServ/LexUriServ.do?uri=OJ:L:2010:276:0033:0079:en:PDF<br>Thus, no ethical approval or guidance was required for this study

Note that full information on the approval of the study protocol must also be provided in the manuscript.

Plants

Seed stocks	No seed stock or plant material was used in this study.
Novel plant genotypes	N/A
Authentication	N/A
